# Use and Performance of Language-based Artificial Intelligence (AI) Models to Screen for Depressive Disorders

**DOI:** 10.1007/s11920-026-01676-2

**Published:** 2026-04-30

**Authors:** Jorge Arias de la Torre, Roman Dahl, Jordi Alonso, Ioannis Bakolis, Lorena Botella-Juan, Alejandro Gonzalez-Diez, Mario F. Juruena, Vicente Martín, Gonzalo Martinez-Alés, Daniel Munblit, Gemma Vilagut, Alex Dregan, Jose M. Valderas

**Affiliations:** 1 Care in Long Term Conditions Research Division, King’s College London, JCMB, Second Floor, Office 2.16. 57 Waterloo Road, London, SE1 8WA UK; 2 Institute of Psychiatry, Psychology and Neurosciences (IoPPN), King’s College London, London, UK; 3 Institute of Biomedicine (IBIOMED), Universidad de León, León, Spain; 4 CIBER Epidemiology and Public Health (CIBERESP), Madrid, Spain; 5 Health Services Research Group, Hospital del Mar Medical Research Institute (IMIM), Barcelona, Spain; 6 Department of Medicine and Life Sciences, Pompeu Fabra University (UPF), Barcelona, Spain; 7 IT Department, Komons Collective SLL, Madrid, Spain; 8 Institute of Psychiatry and Neuroscience (IoPPN), King’s College London and South London and Maudsley (SLaM) NHS Foundation Trust, London, UK; 9 Mount Sinai Hospital, New York, NY USA; 10 CAUSALab, Harvard TH Chan School of Public Health, Boston, MA USA; 11 La Paz Research Institute (IdiPAZ), Madrid, Spain; 12 Network Center for Biomedical Research in Mental Health (CIBERSAM), Madrid, Spain; 13 Department of Paediatrics and Paediatric Infectious Diseases, Institute of Child’s Health, Sechenov University, Moscow, Russia; 14 Research and Clinical Center for Neuropsychiatry, Moscow, Russia; 15 Department of Medicine, National University of Singapore, Singapore, Singapore; 16 Department of Family Medicine, National University Health System, Singapore, Singapore; 17 Centre for Research in Health Systems Performance (CRiHSP), National University of Singapore, Singapore, Singapore

**Keywords:** Depression, Screening, Artificial Intelligence, Natural Language Processing

## Abstract

**Purpose of Review:**

This review summarised and assessed the current evidence on the use of language-based Artificial Intelligence (AI) models for the screening of depressive disorders in adults.

**Recent Findings:**

Most of the studies assessing the use of language-based AI models for the screening of depression were conducted in high-income countries, primarily in the US, and used heterogeneous datasets and populations, limiting generalizability. Most of the evidence was based on text data analysed using transformer models, followed by speech data through convolutional or recurrent neural networks (CNN and RNN models respectively). The evidence combining both modalities (text and speech) and models is limited. Considering the performance for the screening of depressive. disorders, the models tested achieved a performance comparable to standard instruments and cut-off scores, such as the 9-item version of the Patient Health Questionnaire (PHQ-9) with a cut-off of 10 or higher.

**Summary:**

A better understanding of the performance of language-based AI models in real-world settings is required. However, the evidence identified shows that they can be a relevant resource for this screening of depressive disorders and, consequently, for preventing them and reduce their prevalence, burden, and impact at all levels.

## Background

Depressive disorders are one of the most prevalent group of mental disorders and one of the leading causes of disability worldwide [1–4]. According to the Global Burden of Disease Study (GBD) data of 2023, the relevance of depressive disorders as health problems has sharply increased in the last three decades, with an increase in their prevalence of approximately a 37% since 2010. Furthermore, depressive disorders are the second highest cause of Years Lived with Disability (YLDs) among both mental and physical disorders highlighting the relevance of their impact at both the population and individual levels. Thus, the development of strategies for tackling the burden and impact of these disorders is urgently required, and their early detection using screening tools constitute a pivotal resource for it [5, 6].

The use of text- and Speech-Based Artificial intelligence (AI) models such as Natural-Language Processing (NLP) models [7], has been proposed as a relevant resource to improve the effectiveness and efficiency of the screening for depressive disorders due to the self-reported nature of their symptoms [8, 9]. These models are aimed to identify statistical patterns in large datasets at high speed and scale beyond human capability. One relevant type of data included in these large datasets are language data from both spoken and written communication (text and/or speech data). These data are that are usually a direct product of clinical practice and, in general, include relevant information about the patient status and symptoms over time. Additionally, these data could be directly or passively captured in the case that they will be considered relevant [10], and their analysis could be easily automatised. Therefore, the use of language-based AI models for analysing language-based data, would constitute a relevant resource for enhancing the effectiveness and efficiency of the screening of depressive disorders.

It should be noted that previous evidence points to a possible language alteration during depressive episodes, including alterations in the word choice or in the speech rate [11, 12]. Despite the possible relevance of these signals, some of them are rarely considered as they could not be always directly and easily measured, analysed and, in some cases even detected, such as subtle but potentially relevant alterations in word frequency or length. However, these signals could be easily and effectively captured using automatic recording methods and quantified and analysed using language-based AI models already in use for other purposes, such as NLP models [13]. Their application would enable analysing possibly relevant semantic and acoustic features, such as semantic features from written data using contextual embeddings, or pitch contours, speed/pause, frequency and word length from speech data, offering complementary inputs to the information directly collected by the clinician [12, 14, 15]. Thus, the use of language-based AI models would constitute a helpful resource for enhancing the effectiveness for detecting possible depressive disorders of traditional screening strategies based on screening questionnaires, such as the 8- or 9-item versions of the Patient Health Questionnaire (PHQ-8 and PHQ-9, respectively).

Language-based AI models are increasingly used in mental health research and clinical practice [16, 17]. Previous evidence about the detection of possible mental disorders from text data, such as the data captured in clinical interviews, electronic health-records, or social media posts, suggests that deep-learning models (including NLP models) outperform traditional machine-learning approaches across many languages [16, 18]. Additionally, a systematic review mapping studies using AI tools in mental healthcare reported that text-based data could be more useful than acoustic data for the detection of different mental health symptoms but, also, identified gaps in external validation and implementation of the tools [19]. Besides, a recent policy review suggests that the use of language-based AI tools would be helpful to improve the operational efficiency of healthcare systems, such as improving the efficiency of screening, scheduling and risk flagging [20].

Despite the growing evidence about the use of language-based AI models for the screening of depressive disorders and their potential advantages [21–23], there is still a big heterogeneity between studies assessing their suitability. Some studies have focused only on a specific language modality for the assessment of the model performance e.g., just focusing on speech data and other studies relied on very specific samples e.g., university students, compromising both the internal and external validity of their findings [24–27]. Given this heterogeneity, there is a need for summarising current evidence about the use and performance of language-based AI models to detect possible depressive disorders. Such knowledge would be helpful for better understanding how these models are and should be used, as well as for determining their potential relevance as screening tools, and guiding future research and practice using them in real-world settings.

## Objective

The aims of this study are: (1) to summarise and synthesise the current evidence about the uses of language-based AI models for the screening of depressive disorders; and (2) to determine their performance, and potential suitability as screening tools.

## Study Selection and Analysis

### Search Strategy and Selection Criteria 

A systematic review of the literature following the Preferred Reporting Items for Systematic Reviews and Meta-Analyses (PRISMA) guidelines has been conducted [28], and its protocol has been registered in PROSPERO (CRD420251121073). Additionally, the framework suggested by Popay et al. [29] has been followed for reporting the results from the review and synthesising the evidence found. The quality and risk of bias were appraised using the updated Prediction Model Risk Of Bias Assessment Tool (PROBAST + AI), a tool specifically designed to assess the quality, risk of bias, and applicability of AI models [30].

Studies focused on the screening of depression in adults (≥ 18 years), (ii) published in peer-reviewed sources, (iii) in English, (iv) using language-based AI models for detecting depressive disorders were considered for inclusion. Studies not assessing the performance or potential suitability of language-based AI models as screening tools for depression, focused on children or adolescents (≤ 17 years), and considering specific population groups as study sample, such as patients with a specific mental or physical disorder, were excluded. Additionally, studies not assessing the Conference abstracts and non-peer reviewed literature (technical reports, books, social-media information, diaries, and other documents) were excluded due to the lack of guarantees about their validity. Additionally, due to the focus of this study on primary evidence literature reviews were also excluded. However, the primary references included within them were considered.

### Literature Search Process, Data Synthesis and Quality of the Evidence

Five databases (PubMed, Scopus, Embase, MEDLINE and APA PsycINFO) were searched for relevant documents, and the search was performed in August 2025 with no other publication time restriction. The search was structured following the Population, Intervention, Comparison, Outcome (PICO) framework as follows [31]: (P) Adult population; (I) Language-based AI model/NLP model to screen for depressive disorders; (C) Traditional assessment formats (e.g., paper-based questionnaires or face-to-face interviews); (O) Accuracy/performance for the assessment of depression. The full search filters used for each specific database can be found in Appendix 1. Records derived from the searches were imported into Rayyan [32], a digital tool to support deduplication, screening, and eligibility review. Potential duplicates were flagged by the tool and manually reviewed, confirmed, and removed. Titles and abstracts of the records remaining were screened for inclusion and exclusion criteria by two independent reviewers. After the screening, full texts of the remaining records were retrieved and screened to yield the final list of studies included. The same reviewers (JAT and RD) were involved also in the data extraction and quality assessment processes, and a third reviewer was considered to moderated disagreements.

The evidence presented in the studies finally selected after the screening process was extracted and synthesised in evidence tables following the Popay et al. [29] framework and assessed using the PROBAST-AI tool, an update of the original PROBAST tool designed for the assessment of the quality, risk of bias, and applicability of studies using AI methods [30]. Quality and risk of bias are assessed using signalling questions, and applicability considering the participants, data sources used in the studies, and the predictors and outcomes considered in them. The tool considers separately the model development and its evaluation, and it’s divided in 4 sections: (1) general information about the study, (2) signalling questions (16 for model development and 18 for model evaluation; 3) quality (for model development) and risk of bias (for model evaluation), 4) rationale for the overall judgments. Based on the previous information an overall judgment about the quality, risk of bias, and applicability is made considering the following categories “low”, “high”, or “unclear” concern about them [30].

## Finding

**Fig. 1:**
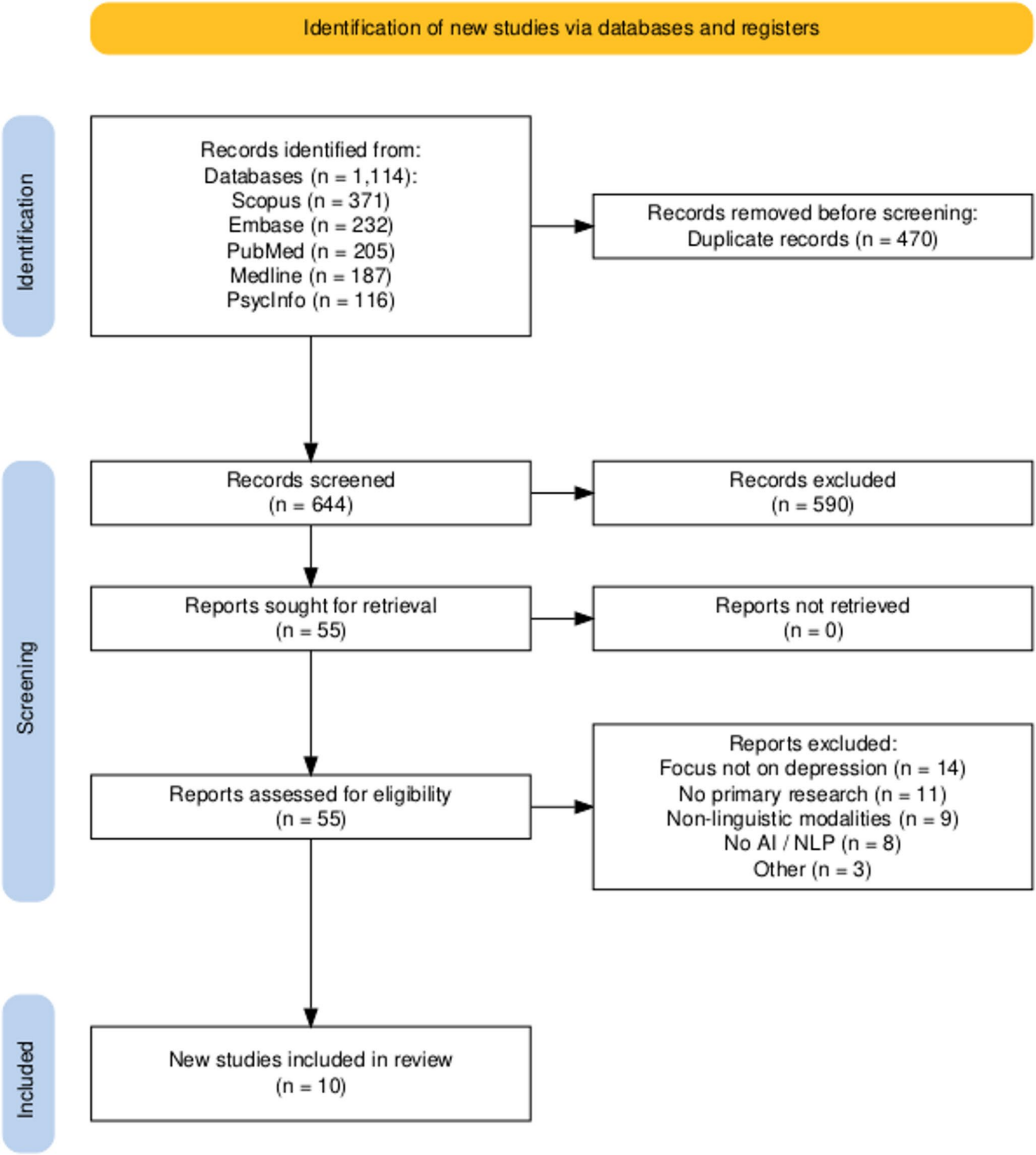
Preferred Reporting Items for Systematic Reviews and Meta-Analyses (PRISMA) flow diagram summarising the literature search. Note: n: number; AI: artificial intelligence; NLP: natural language processing

Figure 1 shows the PRISMA flowchart including detailed information about the literature selection process. The search strategy yielded 1,114 records, of which 470 were identified as duplicates. The remaining 644 records were screened and, from them, 55 were selected for full-text revision. From these documents, 45 were excluded leading to 10 studies that met all the inclusion criteria. All the studies were published in the last 5 years i.e., from 2020 to 2025.

Table 1 shows the general characteristics and main results from the 10 studies meeting the inclusion criteria. The total population included in the studies reviewed was composed of 3,001 adults with a percentage of women ranging from a 38% to a 78%, and a mean age ranging from 21.3 to 81.0 years. In 6 out of the 10 studies included, the age of participants was not directly described. The participants of the 10 studies finally included belong to 5 different countries (6 of the studies were conducted using samples from the US). Besides, in 6 out of the 10 studies the data proceed from research contexts, in 3 studies from clinical contexts (a geriatric clinic, a psychiatric hospital and an academic out-patient centre) and in 1 from self-referrals of university students.

**Table 1:** General characteristics and results from the studies meeting the inclusion criteria

Author (Year)	*N* (%)	Mean age (range)	Country	Study context
Du et al. (2023)	241 (46)	Not reported	US, China	US research interviews; Chinese multi-task speech interview
Lin et al. (2022)	1,698 (63)	Not reported	China	Chinese geriatric clinic; University hospital out-patients
Mao et al. (2023)	189 (46)	Not reported	US	US research interviews
Milintsevich et al. (2023)	189 (46)	Not reported	US	US research interviews
Munthuli et al. (2023)	80 (76)	35.4 (18–63)	Thailand	Bangkok psychiatric hospital
Ohse et al. (2024)	82 (59)	31.6 (18–62)	Germany	Academic out-patient centre
Podina et al. (2023)	773 (66)	21.3 (18–53)	Romania	University self-referrals
Smith et al. (2020)	46 (78)	81.0 (70–93)	US	Assisted-living free-speech & reading (research)
Wang et al. (2025)	275 (38)	Not reported	US	US research interviews.
Zhang et al. (2025)	275 (38)	Not reported	US	US research interviews

*N* Number of participants, % Percentage of women

The general characteristics of the data used in the 10 studies finally included, and the performance of the AI models evaluated in them could be found in Table 2. Considering the datasets used in the different studies, 5 out of 10 used the Distress Analysis Interview Corpus/Wizard-of-Oz (DAIC-WOZ) and its extension the Extended Distress Analysis Interview Corpus (E-DAIC), in one studies combined with the MODMA: Multi-modal Open Dataset for Mental-disorder Analysis. Also, 4 studies collected their own data, and 1 additional study combined the Oizys data and data directly collected for the study. These databases included data in 4 different languages and from 3 data modalities captured in them were analysed. The data modalities were: text-only data analysed in 6 studies (from transcripts only, breaking down words into smaller tokens, creating a numeric context map, and classifying speakers as depressed or not), speech-only data analysed in 3 studies (acoustic pipelines to turn raw speech data into pictures of spectrograms to track tempo, rhythm, and acoustic cues), and multimodal data analysed in 1 study (combining text and speech data). Additionally, 9 studies used as reference measure for determining possible depression the PHQ-8 or PHQ-9 with a cut-off score of 10+, except in the study of Munthuli et al. (2023), in which the cut-off score used was of 9+. Furthermore, 2 studies used the Hamilton Depression Rating Scale (HAM-D) with a cut-off score for depression of 8. One of these studies used it together with the PHQ-9 and the other together with the Mini International Neuropsychiatric Interview – MINI. Regarding the specific models used and their performance for the screening of depression, 6 studies used transformers models, specifically Bidirectional Encoder Representations from Transformers (BERT), Robustly Optimised BERT Approach (RoBERTa), and Cross-Lingual (XLM-R) models. These models showed high values for the AUC and F1 (the harmonic mean of sensitivity and positive-predictive value), ranging between 0.85 and 0.95, and between 0.73 and 0.90, respectively. Speech-only data were analysed in 3 of the studies selected using 2 combinations of acoustic models, Convolutional Neural Network (CNN) + Bidirectional Long Short-Term Memory network models (Bi-LSTM) and Hidden-Unit BERT (HUBERT) + Gated Recurrent Unit models (GRU). These models showed an AUC ranging from 0.87 to 0.90 and a F1 from 0.75 to 0.86, but differences in accuracy according to the language of the data were found. Furthermore, multimodal data was analysed in 1 study using a dual model (CNN + Bi-LSTM). This model showed highest AUC and F1 (both 0.99) for detecting depressive disorders using multimodal data. However, the lack of validation of the results from this study must be noted. Besides, it should be also noted that 8 of the studies tested their algorithms only on data drawn from the same source as they were trained on, and that only 2 gave detailed information about errors in classification with one study providing a calibration curve, and another dissecting false positive and negatives. of them

**Table 2:** General characteristics of the data used in the studies included and performance of the models evaluated

Author (Year)	Dataset used	Language	Data modality	Depression measure	Model family	AUC	F1	EV/ER
Du et al. (2023)	DAIC-WOZ/MODMA	English, Mandarin	Speech	PHQ-8 (DAIC)/PHQ-9 (MODMA)	CNN–RNN hybrid	—	0.75 (DAIC)/86 (MODMA)	No/No
Lin et al. (2022)	Oizys; Own data	Mandarin	Speech	HAM-D; MINI	RNN	0.87	—	Yes/No
Mao et al. (2023)	DAIC-WOZ	English	Speech & text	PHQ-8	CNN–RNN hybrid	0.99	0.99	No/No
Milintsevich et al. (2023)	DAIC-WOZ	English	Text	PHQ-8	TF + RNN hybrid	—	0.74	No/No
Munthuli et al. (2023)	Own data	Thai	Text	PHQ-9; HAM-D	TF	—	0.90	No/No
Ohse et al. (2024)	Own data	German	Text	PHQ-8	TF	—	0.73	No/No
Podina et al. (2023)	Own data	English	Text	PHQ-9	TF	0.85	—	No/No
Smith et al. (2020)	Own data	English	Speech	PHQ-9	ML	0.90	—	Yes/No
Wang et al. (2025)	E-DAIC	English	Text	PHQ-8	TF + CNN hybrid	0.95	0.89	Yes/Yes
Zhang et al. (2025)	DAIC-WOZ; E-DAIC	English	Text	PHQ-8	TF	—	0.88 (DAIC)/0.86 (E-DAIC)	Yes/Yes

*AUC* Area under the curve, *F1* Harmonic mean of sensitivity (recall) and positive-predictive value (precision), *EV*/*ER* External validation/Errors: validation of the results using different data than for its training/reporting specific classification errors. *DAIC*-*WOZ* Distress Analysis Interview Corpus/Wizard-of-Oz, *E*-*DAIC* Extended Distress Analysis Interview Corpus, *MODMA* Multi-modal Open Dataset for Mental-disorder Analysis, *PHQ*-*8* 8-item version of the Patient Health Questionnaire, *PHQ*-*9* 9-item version of the Patient Health Questionnaire, *MINI* Mini International Neuropsychiatric Interview, *HAM*-*D* Hamilton Depression Rating Scale, *CNN* Convolutional neural network, *RNN* Recurrent neural network, *TF* Transformer, *ML* Machine Learning; * Mean age (range) reported for the Oizys dataset: 34 (18–70)

Considering the quality assessment of the studies finally selected, most of them showed the model architecture and features in detail, leading to low risk-of-bias assessments on the PROBAST + AI Predictor domain (Appendix 2). However, none of the studies reported sex-specific analysis or performance, except for one study that reported a no meaningful difference between men and women (an AUC of 0.87 and 0.86 for males and females, respectively). Besides, the 2 studies including the oldest samples did not find meaningful drop in accuracy in older age groups.

## Conclusions and Clinical Implications

This systematic review summarises the current evidence about the use of language-based AI models for the screening of depressive disorders in adults. This evidence suggests that language-based AI models show a suitable performance for detecting possible cases of depressive disorder, similar to traditional screening tools, such as the PHQ-9. Specifically, transformer models using text data show a particularly good performance for detecting possible cases of depressive disorders. Besides, hybrid models using voice data, particularly, on multimodal data showed also promising results as screening tools for depressive disorders. Despite their implementation and use in real-world settings still requires further rigorous research using population-based studies, the evidence available suggests that language-based AI models using text and multimodal data would constitute a relevant resource to screen for depressive disorders.

Regarding the models and data modalities, transformer models using text data consistently reported a suitable performance for detecting possible cases of depression [33–38], matching or surpassing benchmarks reported for the PHQ-8 or PHQ-9 in routine practice [39, 40]. Additionally, models using text and speech data e.g., hybrid models including CNN or RNN models, had also a suitable performance but showed clear deterioration in their accuracy when tested on external data [41–44]. These findings suggest that models using only text data may currently be the best choice for screening of depressive disorders. Besides, in line with previous research [24, 25], our results suggest that models including speech data in addition to text data, such as CNN models based on voice pipelines, would require additional training to be able to cope with different environments, accents, and other linguistic and acoustic features before they can serve busy triage phone lines or primary care clinics.

Considering the validity of the assessments made in the different studies, most of them test their algorithms only on data drawn from the same source that supplied the training material. The three studies that externally tested the validity of the assessments confirmed the well-established risk in AI modelling of over-fitting [45]. Thus, external validation stands out as a relevant missing factor in most of the studies selected, and potentially a bottleneck towards implementation. These results align with the proposal of different authors [46], about the need of using representative data or data from Electronic Health Records (EHRs), and valid measures of depression (e.g., diagnostic codes) for externally validate the models for the screening of depressive disorders and ensure their reliability and, especially, their validity. Furthermore, previous research suggests that combining text- or speech-based models with other automatic analytical approaches based on non-linguistic data (such as machine learning models), improve the reliability and validity of their use alone [7]. Thus, further research aimed to externally validate language-based models using population-based and representative data [47], as well as to assess their performance as screening tools for depression alone and combined with other analytical approaches, will constitute a key step forward for ensuring the validity, reliability and the suitability of their implementation in real-world settings.

The use of screening tools and not clinical interviews (currently considered the gold standard for the assessment of depressive disorders) as reference to determine the accuracy of language-based models should be highlighted. Despite prior evidence pointing out systematically that the use of questionnaire cut-off scores may misclassify possible cases and a sizable share of dysthymia and mixed-anxiety cases [48], all the studies finally included in this review relied, at least partially, on questionnaires using standard cut-off scores (e.g., the PHQ-8 or PHQ-9, and HAM-D) for identifying cases of depressive disorders. Similarly, the misclassification derived from the use of questionnaire cut-off scores has been observed using language-based models [49]. This misclassification would be even higher in the case of these models, as a screening questionnaire has been used as reference to assess the model performance, potentially leading to artificial estimations about the cases identified. Hence, in line with the suggestions of previous research and of the depression screening task forces worldwide [34, 36, 50], further research using clinical labels or diagnostic codes (e.g., from clinical interviews or ICD codes) as reference for the assessment of the performance of screening tools, in this case of language-based AI models, appears pivotal for ensuring their real relevance to support early referral decisions. 

Different limitations of this review should be discussed. The search was only focused on articles in English. Hence, a wider language search would be helpful to increase the number of studies selected and the diversity of their samples. Besides, the limitation related to the heterogeneity of the studies finally included and the geographical scope of the data used in them should be also mentioned. The studies included were heterogeneous in the models used and their methods limiting to certain extent the external validity of the findings as well as their relevance for being meta-analysed. Additionally, it should be mentioned the limitations in relation to the data used in the studies finally included. Two very specific sources (a Mandarin-speaking older adults recorded in Chinese geriatric clinics and English-speaking university students interacting with a Romanian chatbot) provided nearly 80% of the participants in the 10 studies finally included, and six studies used the same datasets including only 275 participants (DAIC-WOZ/E-DAIC). Thus, while the study findings show the promising potential of language-based AI models for the screening of depression, the geographic and linguistic skew of the data analysed in them compromises the external validity of their results and narrows the applicability of the models. Finally, it should be also discussed the limitation about the lack of revision of misclassification errors, made in only two of the studies finally included [33, 34]. These error revisions led to different potentially relevant findings, such as models flagging patients as depressed due to emotionally charged but healthy narratives, or algorithms systematically nudging predicted PHQ-8 scores below the diagnostic threshold due to a lack of depressed cases in the underlying dataset. This highlights the relevance of conducting and reporting error analyses for ensuring the suitability of the models to be implemented in clinical and non-clinical settings.

In conclusion, our review shows that language-based AI models achieve a similar performance for the screening of depressive disorders than self-reported screening questionnaires using standard cut-off scores (e.g., the PHQ-8 or PHQ-9 using a cut-off score of for positives 10+). While promising, the results found indicate that further research using population-wide data and valid measures of depressive disorders (e.g., diagnostic interviews) is required to assess the performance of models, and to determine their suitability to be used for the screening of depression. Therefore, the findings presented underscore the potential relevance and advantages that the use of language-based AI models for the screening of depressive disorders and, consequently, for their early detection, prevention and the reduction of their consequences, burden and impact.

## Key References

- Le Glaz A, Haralambous Y, Kim-Dufor DH, Lenca P, Billot R, Ryan TC, et al. Machine Learning and Natural Language Processing in Mental Health: Systematic Review. J Med Internet Res. 2021; 23(5). 10.2196/15708. **This systematic review offers a general perspective about the use of machine learning and natural language Pprocessing in mental health.**
- He C, Xia Y, Cheung YS, Lam ST, Chen SC, Valderas JM, et al. Electronic patient-reported outcome measures for triaging and scheduling outpatient appointments: a systematic review and meta-analysis. J Am Med Inform Assoc. 2025; 32:1219–26. 10.1093/JAMIA/OCAF078. **This review assessed the effectiveness of use electronic patient-reported outcome measures for screening and making appointments for secondary care in adult patients with chronic medical conditions.**
- Leal SS, Ntalampiras S, Sassi R. Speech-based Depression Assessment: A Comprehensive Survey. IEEE Trans Affect Comput [Internet]. 2024;1–16. 10.1109/TAFFC.2024.3521327. **This manuscript explores the use of speech data for the assessment of depression.**
- Tornero-Costa R, Martinez-Millana A, Azzopardi-Muscat N, Lazeri L, Traver V, Novillo-Ortiz D. Methodological and quality flaws in the use of artificial intelligence in mental health research: systematic review. JMIR Ment Health. 2023; 10:e42045. 10.2196/42045. **This systematic review reflects on the use of AI tools in mental health research and shows possible ways of using it**,** including for the screening of mental disorders.**

## Data Availability

No datasets were generated or analysed during the current study.

## Appendix 1. Search strategy by database

**PubMed**

(text* or speech*)

AND (“artificial intelligence“[MeSH] OR “artificial intelligence” OR a.i. OR “natural language processing” [MeSH] OR NLP)

AND (triage[MeSH] OR diagnosis[MeSH] OR triag* OR diagnos* OR screen*)

AND (“depressive disorder“[MeSH] OR depress*)

AND (accura* OR detect* OR perform*)

**SCOPUS**

TITLE-ABS-KEY(text” or speech*)

AND TITLE-ABS-KEY(“artificial intelligence” OR a.i. OR “natural language processing” OR NLP)

AND TITLE-ABS-KEY(screen* OR diagnos* OR triag*)

AND TITLE-ABS-KEY(depress*)

AND TITLE-ABS-KEY(accura* OR detect* OR perform*)

**Embase (Ovid)**

1. (text* or speech*).mp.
2. exp artificial intelligence/or artificial intelligence.mp. or ai.mp. or exp natural language processing/or natural language process*.mp. or nlp.mp.
3. exp patient triage/or triag*.mp. or exp screening/or screen*.mp. or exp diagnosis/or diagnos*.mp.
4. exp depression/or depress*.mp.
5. exp accuracy/or accura*.mp. or detect*.mp. or exp performance/or perform*.mp.
6. 1 and 2 and 3 and 4 and 5

**Medline (Ovid)**

1. (text* or speech*).mp.
2. exp artificial intelligence/or artificial intelligence.mp. or ai.mp. or exp natural language processing/or natural language process*.mp. or nlp.mp.
3. exp triage/or triag*.mp. or exp screening/or screen*.mp. or exp diagnosis/or diagnos*.mp.
4. exp depression/or depress*.mp.
5. (accura* or detect* or perform*).mp.
6. 1 and 2 and 3 and 4 and 5.

**APA PsycInfo (Ovid)**

1. (text* or speech*).mp.
2. exp artificial intelligence/or artificial intelligence.mp. or ai.mp. or exp natural language processing/or natural language process*.mp. or nlp.mp.
3. exp triage/or triag*.mp. or exp screening/or screen*.mp. or exp diagnosis/or diagnos*.mp.
4. exp depression/or depress*.mp.
5. (accura* or detect* or perform*).mp.
6. 1 and 2 and 3 and 4 and 5

## Appendix 2.

Risk of Bias Assessment according to the updated Prediction Model Risk Of Bias Assessment Tool (PROBAST+AI).

**Table:**

Study (year)	Participants	Predictors	Outcome	Analysis	Overall
Du et al. (2023)	H	L	H	H	**H**
Lin et al. (2022)	L	L	L	U	**L**
Mao et al. (2023)	H	L	H	H	**H**
Milintsevich et al. (2023)	L	H	H	H	**H**
Munthuli et al. (2023)	L	L	L	H	**L**
Ohse et al. (2024)	L	L	H	H	**U**
Podina et al. (2023)	L	L	H	H	**U**
Smith et al. (2020)	H	L	H	H	**H**
Wang et al. (2025)	L	L	H	L	**L**
Zhang et al. (2025)	L	U	H	H	**U**

*H* high risk, *L* low risk, *U* unclear

## References

[1] The Lancet Psychiatry. Global Burden of Disease 2021: mental health messages. Lancet Psychiatry [Internet]. Elsevier Ltd. 2024;11:573. 10.1016/S2215-0366(24)00222-0 10.1016/S2215-0366(24)00222-0 39025623

[2] Arias-de la Torre J, Vilagut G, Ronaldson A, Bakolis I, Dregan A, Martín V et al. Prevalence and variability of depressive symptoms in Europe: update using representative data from the second and third waves of the European Health Interview Survey (EHIS-2 and EHIS-3). Lancet Public Health [Internet]. Elsevier Ltd. 2023;8:e889–98. 10.1016/S2468-2667(23)00220-7 10.1016/S2468-2667(23)00220-7 37898521

[3] Moreno-Agostino D, Wu YT, Daskalopoulou C, Hasan MT, Huisman M, Prina M, et al. Global trends in the prevalence and incidence of depression: a systematic review and meta-analysis. J Affect Disord. 2021;281:235–43. 10.1016/j.jad.2020.12.035. 33338841 10.1016/j.jad.2020.12.035

[4] GBD 2023 Disease and Injury and Risk Factor Collaborators, Elsevier. Burden of 375 diseases and injuries, risk-attributable burden of 88 risk factors, and healthy life expectancy in 204 countries and territories, including 660 subnational locations, 1990–2023: a systematic analysis for the Global Burden of Disease Study 2023. Lancet. 2025;406:1873–922. 10.1016/S0140-6736(25)01637-X. 41092926 10.1016/S0140-6736(25)01637-X PMC12535840

[5] Kisely S, Scott A, Denney J, Simon G, Br J Psychiatry. Duration of untreated symptoms in common mental disorders: association with outcomes: International study. Br J Psychiatry. 2006;189:79–80. 10.1192/BJP.BP.105.019869. 16816310 10.1192/bjp.bp.105.019869

[6] Arias de la Torre J, Ronaldson A, Vilagut G, Martínez-Alés G, Dregan A, Bakolis I, et al. Implementation of community screening strategies for depression. Nat Med. 2024;30(4):930–2. 10.1038/S41591-024-02821-1. 38413728 10.1038/s41591-024-02821-1

[7] Le Glaz A, Haralambous Y, Kim-Dufor DH, Lenca P, Billot R, Ryan TC, et al. Machine learning and natural language processing in mental health: systematic review. J Med Internet Res. 2021;23(5):e15708. 10.2196/15708. 33944788 10.2196/15708 PMC8132982

[8] Kalusivalingam AK, Sharma A, Patel N, Singh V. Enhancing Mental Health Diagnostics: Implementing Convolutional Neural Networks and Natural Language Processing in AI-Based Assessment Tools. Int J AI ML [Internet]. 2012;1. https://cognitivecomputingjournal.com/index.php/IJAIML-V1/article/view/127

[9] He C, Xia Y, Cheung YS, Lam ST, Chen SC, Valderas JM, et al. Electronic patient-reported outcome measures for triaging and scheduling outpatient appointments: a systematic review and meta-analysis. J Am Med Inform Assoc. 2025;32:1219–26. 10.1093/JAMIA/OCAF078. 40407342 10.1093/jamia/ocaf078 PMC12202323

[10] Ramanarayanan V. Multimodal technologies for remote assessment of neurological and mental health. J Speech Lang Hear Res. 2024;67:4233–45. 10.1044/2024_JSLHR-24-00142. 38984943 10.1044/2024_JSLHR-24-00142 PMC12379571

[11] Cummins N, Scherer S, Krajewski J, Schnieder S, Epps J, Quatieri TF. A review of depression and suicide risk assessment using speech analysis. Speech Commun. 2015;71:10–49. 10.1016/j.specom.2015.03.004.

[12] Leal SS, Ntalampiras S, Sassi R. Speech-based Depression Assessment: A Comprehensive Survey. IEEE Trans Affect Comput [Internet]. 2024. 10.1109/TAFFC.2024.3521327. 1–16.

[13] Low LSA, Maddage NC, Lech M, Sheeber LB, Allen NB. Detection of clinical depression in adolescents’ speech during family interactions. IEEE Trans Biomed Eng. 2011;58:574–86. 10.1109/TBME.2010.2091640. 21075715 10.1109/TBME.2010.2091640 PMC3652557

[14] Aloshban N, Esposito A, Vinciarelli A. What you say or how you say it? Depression detection through joint modeling of linguistic and acoustic aspects of speech. Cognit Comput. 2022;14:1585–98. 10.1007/s12559-020-09808-3.

[15] Morales MR, Levitan R. Speech vs. text: A comparative analysis of features for depression detection systems. 2016 IEEE Spoken Language Technology Workshop (SLT) [Internet]. 2016;136–43. 10.1109/SLT.2016.7846256

[16] Tornero-Costa R, Martinez-Millana A, Azzopardi-Muscat N, Lazeri L, Traver V, Novillo-Ortiz D, et al. Methodological and quality flaws in the use of artificial intelligence in mental health research: systematic review. JMIR Ment Health. 2023;10:e42045. 10.2196/42045. 36729567 10.2196/42045 PMC9936371

[17] Dehbozorgi R, Zangeneh S, Khooshab E, Nia DH, Hanif HR, Samian P, et al. The application of artificial intelligence in the field of mental health: a systematic review. BMC Psychiatry. 2025;25(1):1–20. 10.1186/S12888-025-06483-2. 39953464 10.1186/s12888-025-06483-2 PMC11829440

[18] Ding Z, Wang Z, Zhang Y, Cao Y, Liu Y, Shen X, et al. Trade-offs between machine learning and deep learning for mental illness detection on social media. Sci Rep. 2025;15(1):1–14. 10.1038/s41598-025-99167-6. 40281061 10.1038/s41598-025-99167-6 PMC12032126

[19] Malgaroli M, Hull TD, Zech JM, Althoff T, Springer Nature. Natural language processing for mental health interventions: a systematic review and research framework. Transl Psychiatry. 2023;13:1–17. 10.1038/S41398-023-02592-2. 37798296 10.1038/s41398-023-02592-2 PMC10556019

[20] Dawoodbhoy FM, Delaney J, Cecula P, Yu J, Peacock I, Tan J, et al. AI in patient flow: applications of artificial intelligence to improve patient flow in NHS acute mental health inpatient units. Heliyon. 2021;7:e06993. 10.1016/j.heliyon.2021.e06993. 34036191 10.1016/j.heliyon.2021.e06993 PMC8134991

[21] Abd-Alrazaq A, AlSaad R, Shuweihdi F, Ahmed A, Aziz S, Sheikh J. Systematic review and meta-analysis of performance of wearable artificial intelligence in detecting and predicting depression. NPJ Digit Med. 2023;6:84. 10.1038/s41746-023-00828-5. 37147384 10.1038/s41746-023-00828-5 PMC10163239

[22] Dharma EM, Prabowo H, Trisetyarso A, Wiguna T. The use of artificial intelligence to predict depression through thermal imaging. AIP Conf Proc [Internet]. 2023;2872:040002. 10.1063/5.0163192.

[23] Yin S-Q, Li Y-H. Advancing the diagnosis of major depressive disorder: integrating neuroimaging and machine learning. World J Psychiatry. 2025. 10.5498/WJP.V15.I3.103321. 40109992 10.5498/wjp.v15.i3.103321 PMC11886342

[24] Otero-González I, Pacheco-Lorenzo MR, Fernández-Iglesias MJ, Anido-Rifón LE. Conversational agents for depression screening: a systematic review. Int J Med Inform. 2024;181:105272. 10.1016/j.ijmedinf.2023.105272. 37979500 10.1016/j.ijmedinf.2023.105272

[25] Smrke U, Mlakar I, Lin S, Musil B, Plohl N, JMIR Mental Health. Language, speech, and facial expression features for artificial intelligence-based detection of cancer survivors’ depression: scoping meta-review. JMIR Ment Health. 2021;8:e30439. 10.2196/30439. 34874883 10.2196/30439 PMC8691410

[26] DeSouza DD, Robin J, Gumus M, Yeung A. Natural Language Processing as an Emerging Tool to Detect Late-Life Depression. Front Psychiatry [Internet]. Frontiers Media S.A. 2021;12:719125. 10.3389/FPSYT.2021.719125/BIBTEX 10.3389/fpsyt.2021.719125 PMC8450440 34552519

[27] Ahmad Wani M, Elaffendi MA, Shakil KA, Shariq Imran A, Abd El-Latif AA. Depression screening in humans with AI and deep learning techniques. IEEE Trans Comput Soc Syst. 2023;10:2074–89. 10.1109/TCSS.2022.3200213.

[28] Page MJ, McKenzie JE, Bossuyt PM, Boutron I, Hoffmann TC, Mulrow CD, et al. The PRISMA 2020 statement: an updated guideline for reporting systematic reviews. BMJ. 2021;372:n71. 10.1136/bmj.n71. 33782057 10.1136/bmj.n71 PMC8005924

[29] Popay J, Roberts H, Sowden A, Petticrew M, Arai L, Rodgers M et al. Guidance on the conduct of narrative synthesis in systematic reviews. A product from the ESRC methods programme Version [Internet]. 2006;1:b92. 10.13140/2.1.1018.4643.

[30] Moons KGM, Damen JAA, Kaul T, Hooft L, Andaur Navarro C, Dhiman P, et al. PROBAST + AI: an updated quality, risk of bias, and applicability assessment tool for prediction models using regression or artificial intelligence methods. BMJ. 2025;388:e082505. 10.1136/bmj-2024-082505. 40127903 10.1136/bmj-2024-082505 PMC11931409

[31] Schardt C, Adams MB, Owens T, Keitz S, Fontelo P. Utilization of the PICO framework to improve searching PubMed for clinical questions. Volume 7. BioMed Central Ltd. 2007;1–6. 10.1186/1472-6947-7-16/TABLES/2. BMC Med Inform Decis Mak [Internet]. 10.1186/1472-6947-7-16 PMC1904193 17573961

[32] Ouzzani M, Hammady H, Fedorowicz Z, Elmagarmid A. Rayyan-a web and mobile app for systematic reviews. Syst Rev BioMed Cent Ltd. 2016;5. 10.1186/S13643-016-0384-4. 10.1186/s13643-016-0384-4 PMC5139140 27919275

[33] Wang N, Kamil R, Al-Haddad SAR, Ibrahim N, Zhao Z, Enhancing AI. Depression Detection Using Transfer Learning. Contemporary Mathematics (Singapore) [Internet]. 2025;6:3054–80. 10.37256/cm.6320256184

[34] Zhang X, Li C, Chen W, Zheng J, Li F. Optimizing depression detection in clinical doctor-patient interviews using a multi-instance learning framework. Sci Rep. 2025;15:6637. 10.1038/s41598-025-90117-w. 39994325 10.1038/s41598-025-90117-w PMC11850819

[35] Milintsevich K, Sirts K, Dias G. Towards automatic text-based estimation of depression through symptom prediction. Brain Inform. 2023;10:4. 10.1186/s40708-023-00185-9. 36780049 10.1186/s40708-023-00185-9 PMC9925661

[36] Ohse J, Hadžić B, Mohammed P, Peperkorn N, Danner M, Yorita A, et al. Zero-shot strike: testing the generalisation capabilities of out-of-the-box LLM models for depression detection. Comput Speech Lang. 2024;88:101663. 10.1016/j.csl.2024.101663.

[37] Munthuli A, Pooprasert P, Klangpornkun N, Phienphanich P, Onsuwan C, Jaisin K, et al. Classification and analysis of text transcription from Thai depression assessment tasks among patients with depression. PLoS One. 2023;18:e0283095. 10.1371/journal.pone.0283095. 36996118 10.1371/journal.pone.0283095 PMC10062633

[38] Podina IR, Bucur A-M, Fodor L, Boian R. Screening for common mental health disorders: a psychometric evaluation of a chatbot system. Behav Inf Technol. 2023. 10.1080/0144929X.2023.2275164.

[39] Arias de la Torre J, Vilagut G, Ronaldson A, Valderas JM, Bakolis I, Dregan A, et al. Reliability and cross-country equivalence of the 8-item version of the Patient Health Questionnaire (PHQ-8) for the assessment of depression: results from 27 countries in Europe. The Lancet Regional Health - Europe. 2023. 10.1016/j.lanepe.2023.100659. 10.1016/j.lanepe.2023.100659 PMC10272490 37332385

[40] Kroenke K, Spitzer RL, Williams JBW. The PHQ-9. J Gen Intern Med. 2001;16:606–13. 10.1046/j.1525-1497.2001.016009606.x. 11556941 10.1046/j.1525-1497.2001.016009606.x PMC1495268

[41] Du M, Liu S, Wang T, Zhang W, Ke Y, Chen L, et al. Depression recognition using a proposed speech chain model fusing speech production and perception features. J Affect Disord. 2023;323:299–308. 10.1016/j.jad.2022.11.060. 36462607 10.1016/j.jad.2022.11.060

[42] Lin Y, Liyanage BN, Sun Y, Lu T, Zhu Z, Liao Y, et al. A deep learning-based model for detecting depression in senior population. Front Psychiatry. 2022. 10.3389/fpsyt.2022.1016676. 36419976 10.3389/fpsyt.2022.1016676 PMC9677587

[43] Smith M, Dietrich BJ, Bai E-W, Bockholt HJ. Vocal pattern detection of depression among older adults. Int J Ment Health Nurs. 2020;29:440–9. 10.1111/inm.12678. 31811697 10.1111/inm.12678

[44] Mao K, Zhang W, Wang DB, Li A, Jiao R, Zhu Y, et al. Prediction of depression severity based on the prosodic and semantic features with bidirectional LSTM and time distributed CNN. IEEE Trans Affect Comput. 2023;14:2251–65. 10.1109/TAFFC.2022.3154332.

[45] Aliferis C, Simon G, Aliferis C, Simon G. Artificial Intelligence (AI) and Machine Learning (ML) for Healthcare and Health Sciences: The Need for Best Practices Enabling Trust in AI and ML. Health Informatics [Internet]. Springer, Cham 2024;1–31. 10.1007/978-3-031-39355-6_1. 39836828

[46] Van Calster B, Steyerberg EW, Wynants L, Van Smeden M. There is no such thing as a validated prediction model. BMC Med. 2023;21:70. 36829188 10.1186/s12916-023-02779-w PMC9951847

[47] Riley RD, Ensor J, Snell KIE, Archer L, Whittle R, Dhiman P, et al. Importance of sample size on the quality and utility of AI-based prediction models for healthcare. Lancet Digit Health. 2025;7:100857. 10.1016/j.landig.2025.01.013. 40461350 10.1016/j.landig.2025.01.013

[48] Arias-de la Torre J, Vilagut G, Ronaldson A, Serrano-Blanco A, Alonso J, Elsevier Ltd. PHQ-8 scores and estimation of depression prevalence – author’s reply. Lancet Public Health. 2021;6:e794. 10.1016/S2468-2667(21)00226-7. 34756167 10.1016/S2468-2667(21)00226-7

[49] Hadzic B, Mohammed P, Danner M, Ohse J, Zhang Y, Shiban Y, et al. Enhancing early depression detection with AI: a comparative use of NLP models. SICE Journal of Control, Measurement, and System Integration. 2024;17:135–43. 10.1080/18824889.2024.2342624.

[50] Wang Y, Han X, Li C, Luo L, Yin Q, Zhang J, et al. Impact of gold-standard label errors on evaluating performance of deep learning models in diabetic retinopathy screening: nationwide real-world validation study. J Med Internet Res. 2024. 10.2196/52506. 10.2196/52506 PMC11358665 39141915

